# Evaluation of Antinociceptive Effect of Pregabalin in Mice and its Combination with Tramadol using Tail Flick Test

**Published:** 2013

**Authors:** Fariborz Keyhanfar, Manzumeh Shamsi Meymandi, Gholamreza Sepehri, Ramin Rastegaryanzadeh, Gioia Heravi

**Affiliations:** a Pharmacology Department., Iran University of Medical Sciences, Tehran, Iran.; b Kerman Neuroscience Research Center, Kerman University of Medical Sciences, Kerman, Iran.

**Keywords:** Pregabalin, Tramadol, Combination, Antinociception, Tail flick

## Abstract

The development of combination therapy is a coherent approach in severe pain treatment. The present study investigated the antinociceptive effect of pregabalin alone and in combination with tramadol in acute pain modeling. Therefore, three groups of male mice received either pregabalin (1 to 400 mg/Kg), tramadol (10 to 80 mg/Kg) or their combination intraperitoneally. Then latency time, maximum possible effect (%MPE) and area under curve (AUC) were calculated in tail flick test. The antinociceptive indexes were significantly increasedin10, 100 and 200 mg/kg ofpregabalin while tramadol showed dose-dependentantinociception (effective dose 50% was 54 to 79 mg/Kg). The antinociceptive effect of 100 mg/Kg of pregabalin (%MPE = 35±4%) was similar to that of 50 mg/Kg of tramadol. The combination of non-analgesic doses (10 mg/Kg) of tramadol and pregabalin did not increase %MPE and AUC, but the co-administration of 30 mg/Kg of tramadol with pregabalin (10 mg/Kg) increased all antinociceptive indexes significantly compared to the controls and with each drug alone.

In conclusion, pregabalin showed a comparable antinociceptive effect to tramadol. The increase in analgesic effect was observed after the combination of low analgesic doses of tramadol with pregabalin, while the combination of non-analgesic doses of each drug reversed the interaction to antagonism. Therefore to increase the analgesic effect in pain management, more attention should be paid to respecting right proportion of drug combination. Further studies that specify the mechanism(s) and statement of interaction are needed to expand these findings to clinical applications.

## Introduction

The development of effective analgesics with fewer side effects and appropriate combination therapy is one of the most important objectives of drug researches. Therefore, new drug classes and combination therapy have been proposed in pain treatment.

Pregabalin known as CI-1008 or S(+)-3-isobutylgaba is a novel anticonvulsant of second generation of gabapentinoids that exerts analgesic effect ofits own or as adjuvant in different models of pain (1-3). Pregabalin has been proposed in particular for pain pharmacotherapy of difficult treating syndromes (4-5) and in clinical neuropathic pain treatment (1,4-6). Pregabalin as an adjuvant interacts synergistically with naproxen to reverse hyperalgesia (7); it increases the efficacy of celecoxib in the treatment of low back pain (8) and reduces pain and opioid consumption after surgery (1,9-10).Tramadol as an atypical opioid is another analgesic used widely in the treatment of moderate to severe pain. Unlike typical opioids, tramadol does not produce significant respiratory depression, constipation and sedation. Moreover, it has low potential for dependency, tolerance and drug abuse. However, tramadol may cause nausea, vomiting and tiredness (11-12). Pregabalin, despite of its high efficacy in neuropathic pain, shows minor side effects such as constipation, transient dizziness, visual disturbance and euphoria (4-6,10).

Furthermore, owing to the favorable pharmacokinetic parameters, combination of tramadol and pregabalin can be considered as desirable in pain treatment. These drugs have common characteristics; both are orally active, hold adequate volume of distribution and cross blood brain barriers (10,13-14). Tramadol protein binding is 20%, while pregabalin does not bind to plasma proteins (10,14). These parameters assign pregabalin as a drug holding minimal probability for drug interactions (10,14).Thus it can be added to tramadol in cases of inadequate pain relief. Moreover, the other advantage of this combination is beneficial anticonvulsive effect of pregabalin that can cover a less frequent side effect of tramadol which occurred also with recommended doses (15). However, the efficacy of this combination in acute pain treatment has not been reported yet.

Thus, the current study was designed to establish antinociceptive effect of pregabalin alone and in combination with tramadol in acute model of pain using tail flick test in mice. 

## Experimental

*Animals*

A total of 150 adult male NMRI mice weighing 25 - 35 g were used. Animals were housed in 4 or 5 mice per cage at a controlled temperature (22 ± 2°C) and 12 h light-dark cycle with free access to food and water. The experiments were carried out during light cycle between 12:00 to 16:00 PM. All animals were used only for one procedure and were humanely sacrificed under anesthesia after the completion of experiment. Animal care was conformed to the guidelines on the study of pain in conscious animals established by the institutional guidelines. Ethical approval for this study (N° 90/141) was provided by local Ethical Committee of Kerman University of Medical Sciences according to the Guide for the Use of Laboratory Animals.

*Drugs*

The drugs used were: Pregabalin (Hetero Drugs Limited, India) and Tramadol (Kamud Drugs, India). All drugs were freshly dissolved in normal saline and were injected at volume of 0.1 mL/10g body weight of mice intraperitoneally (IP). 

*Antinociception measurement*

Tail flick test as an acute model of pain was used to assess the antinociceptive effect of the drugs by measuring the latency of response. Radiant heat was applied to the tail at 5-8 cm from the tip using a tail flick apparatus (PANLAB 7160, Spain). Tail flick latency time was measured as the time from the onset of the heat exposure to the withdrawal of the tail. The intensity of radiant heat was adjusted to yield the baseline latencies of 2-4 sec. The heat stimulus was discontinued after 10 sec to avoid tissue damages (Cut off point =10 sec). 

The animals were allowed to habituate to laboratory surroundings before tail flick test and the investigator was blind to the drug injected. For each animal, baseline latency was obtained as the mean of three measurements before the administration of each drug. The animals showing values of baseline latency time less than 2 or more than 4 sec were excluded from the study. The remnant mice were randomly assigned to experimental groups of eight mice. The latency times were determined in 15 min intervals for 90 min from the time of drug or normal saline injection. 

Time course of antinociceptive response of individual drug was constructed by plotting the mean of latency times as a function of time. Antinociception was quantified as either tail flick latency time or percentage of maximal possible effect (%MPE) or the area under curve (AUC) which includes both maximum effects and duration of action (16). The %MPE was calculated as [(T_1 _- T_0_)/(T_2 _- T_0_)]×100. T_0 _and T_1 _were the tail flick latencies before and after drug administration, and T_2_ was the cut off time.

The AUC was calculated considering Tail Flick Latency time (TFL) from 15 to 90 post-injection based on Trapezoid rules (16-17) as follows: AUC=15×TFL[(MIN 15)+(MIN 30)+(MIN 45)+(MIN 60)… +(MIN 90)/2].

*Procedure*

In order to determine the antinociceptive effect of tramadol and pregabalin, the treated groups of eight mice received tramadol at the doses of 10, 20, 40 and 80 mg/Kgand pregabalin at doses of 1, 10, 100, 200 and 400 mg/Kg (IP) respectively, while control group received normal saline. The chosen doses were aimed to cover the maximum possible range of antinociceptive effect. The dose range of tramadol was selected based on previous studies (18-20), while for pregabalin as a new compound in tail flick procedure,we referred to other animal models of pain assessment (21-22). However, the doses of more than 200 mg/Kg were never used before. Then based on latency times, the AUC and %MPE were calculated for each group. The %MPE at the 60^th^ and 75^th^ minutes were the highest ones in the course of study, which were used for further comparisons. 

To determine the ED_50 _(estimated dose to produce 50% of antinociception), %MPE value at the point of time when greatest antinociception was observed were used to built dose-response curve. So the doses were plotted on X-axis of the Cartesian system of coordinate while the corresponding %MPE was plotted on Y-axis. Moreover, both values were analyzed via least-square linear regression analysis according to Motulsky and Christopoulos (23).

The evaluation of nociceptive effect of the co-administration of both drugs was exhibited using non-analgesic, low-analgesic and analgesic doses of tramadol (10, 30 and 50 mg/Kg) with minimum effective dose of pregabalin (10 mg/Kg). Afterward, the highest %MPE or %MPE_60_ of combination groups was compared to those of the control group in order to determine their antinociceptive effect. Also, %MPE_60_ ofeach combination group was compared to that of respective individual drugs to determine the effect of drug interaction.

*Statistics*

Results were expressed as mean ± SEM of eight mice except for the control group (n = 12) in combinational comparisons. Two way repeated measures of Analysis of Variance (ANOVA) was used to assess the effect of dose, time and their interaction in time- response curve of each drug during seven consecutive (0, 15^th^, 30^th^, 45^th^, 60^th^ ,…) measurements of latency times (time course). In this model, the dependent variable was latency time or %MPE and latency time before the injection was considered as covariate. One way analysis of variance determined the differences of %MPE or AUC among independent treated groups and the post-hoc used was Tukey test. ED_50_ values with 95% confidence interval were calculated by linear regression using GraphPad Prism software version 5 (GraphPad Software Inc., San Diego, CA, USA). Statistical analysis was done using SPSS software version 11.5 (Chicago, Illinois, USA). A value of p < 0.05 was considered significant. 

## Results

*Antinociceptive effect of tramadol alone*

The antinociceptive effect of tramadol showed increasing trend at all doses (10, 20, 40 and 80 mg/Kg) and maximum antinociception was observed after injection of 80 mg/Kg at the 75^th^ minute (%MPE_75_=88.4±4.8%).

**Figure 1:**
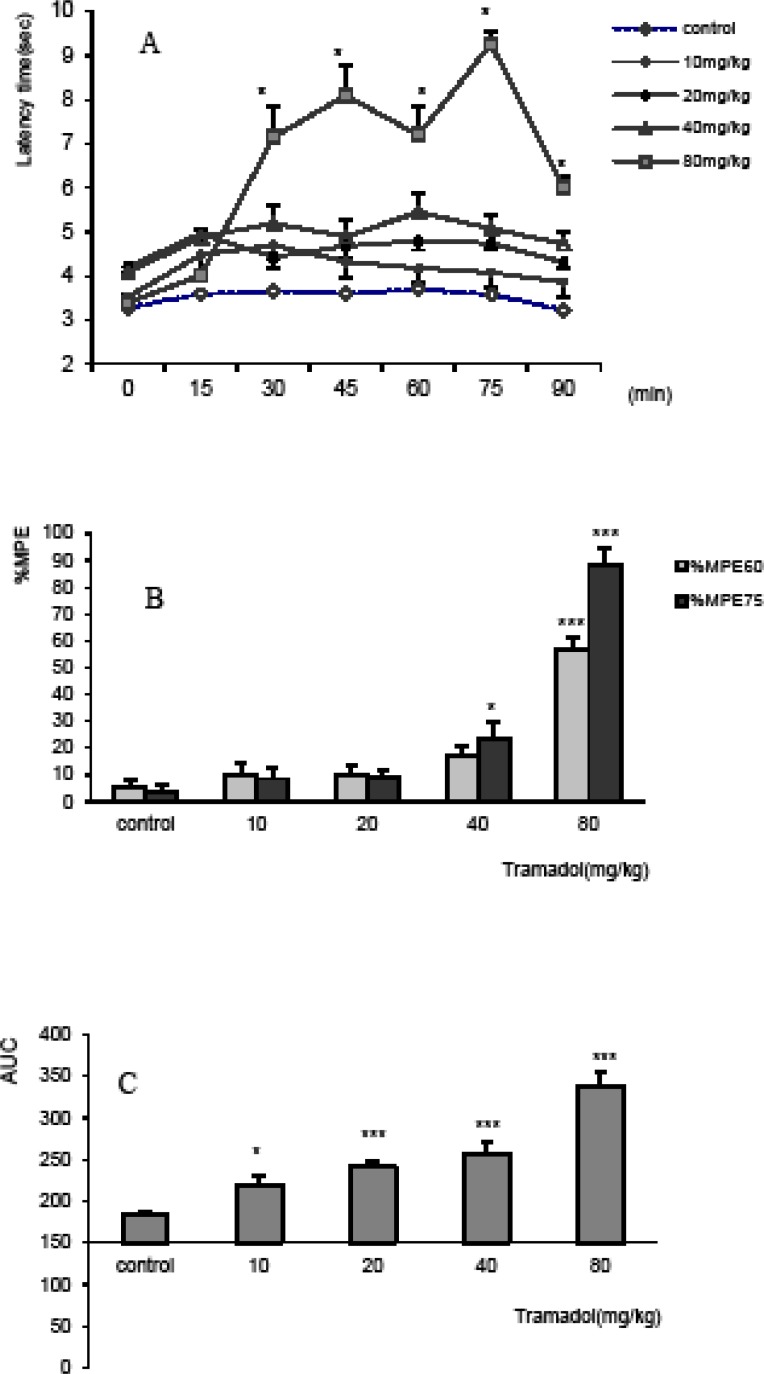
The antinociceptive effect of tramadol in tail flick test, expressed as A) Time-response curve, B) Maximum possible effect (%MPE) at 60^th^ and 75^th^ min, and C) Area under curve (AUC). The mice received intraperitoneally, normal saline and tramadol at doses of 10, 20, 40 and 80 mg/Kg. A). The variation of latency times of different doses during 90 min of the experiment showed similar pattern and temporal variation, however, the interaction of dose showed latency time’s increase by dose. The time response curve of tramadol at dose of 80 mg/Kg was significantly increased compared to the control and all other doses. *p < 0.05 compared to all other doses and p < 0.001 compared to the control. B) %MPE_60_ and %MPE_75_ were increased in dose-dependent manner. *p < 0.05 and ***p < 0.005 compared to controls and all other doses. C) AUC increased in a dose-dependent manner. *p < 0.05 and ***p < 0.005 compared to control. Data were expressed as mean ± SEM of 8 mice

Based on the results of repeated ANOVA model, the temporal variations and the pattern of different doses of tramadol were not different (F_5,185_=0.88, p=0.5), while the interaction between time and dose resulted in significant difference (F_20,185_=13.1, p=0.000). The comparison of the variations of latency times of different doses of tramadol in the course of tail-flick test showed that the time course of response of all doses of tramadol (10 mg/Kg; p < 0.05 and 20, 40, 80 mg/Kg; p < 0.005) was significantly increased compared to control group (Figure 1A).

**Figure 2:**
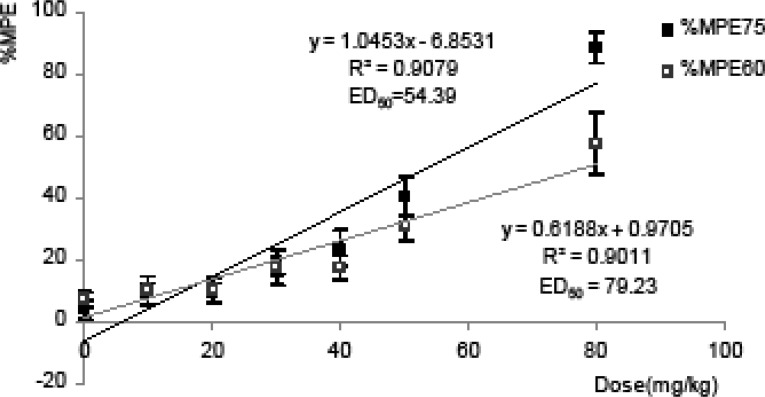
Dose-response relationship between various doses of tramadol and their %MPE using tail flick in mice. %MPE at75^th^ and 60^th^ min after intraperitoneal injection of eight doses of tramadol and respective doses were plotted into Cartesian system of coordinate and analyzed with least-squares linear regression. Data were as mean of %MPE ± SEM. of at least 6 mice

The antinociceptive effect in term of %MPE showed significant differences among doses injected just thirty minutes after the experiment (p < 0.001; data non shown). However, %MPE was much more significant at the 60^th^ and 75^th^ minutes and showed a dose-dependent relationship (Figure2). Thereby, %MPE_60_ and %MPE_75_ of 80 mg/Kgof tramadol increased significantly compared to the control values and all other doses (p < 0.005), while %MPE_75_ of 40 mg/Kgsolely increased compared to the respective control group (p < 0.05) (Figure 1B). 

Area under curve as well as %MPE_75_ showed dose-response relationship. AUC of control group was significantly less than that of tramadol at doses of 10, 20, 40 and 80 mg/Kg (p < 0.05 for dose of 10 mg/Kg and p<0.005 for all others) (Figure 1C). 

In Figure 2, the linear regression of respective dependent variable %MPE_75_ and %MPE_60 _showed a dose-dependent relationship (R^2^> 0.9). Based on this linear dose response curve, the calculated ED_50_ with 95% confidence intervals were 44.34 to 71.02 and 64.03 to 110.2 mg/Kg. Therefore due to the wide variability of ED_50_ calculated in these models, we used doses of 10, 30 and 50 mg/Kg of tramadol in studying combination groups.

*Antinociceptive effect of pregabalin alone*

**Figure 3:**
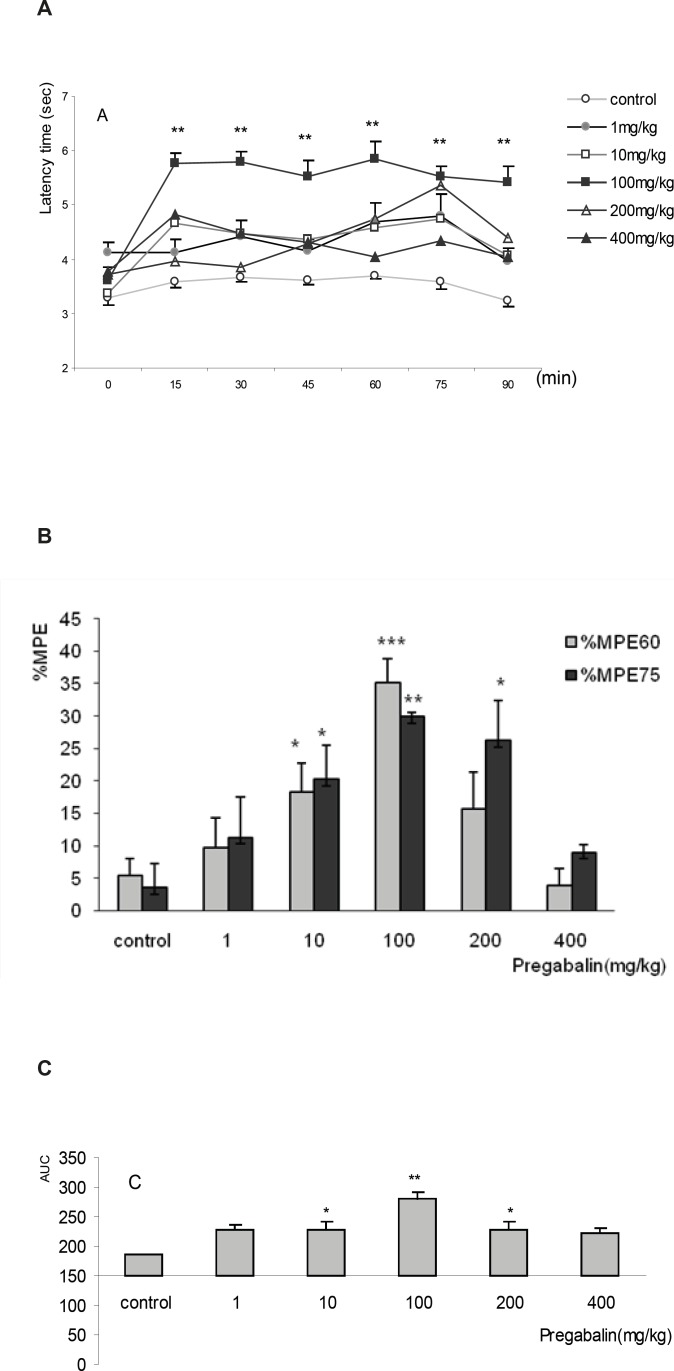
The antinociceptive effect of pregabalin in tail flick test, expressed as A) Time-response curve, B) Maximum possible effect (%MPE) at 60^th ^and 75^th^ min , and C) Area under curve (AUC). The mice received intraperitoneally, normal saline and pregabalin at the doses of 1, 10, 100, 200 and 400 mg/Kg A) The variation of latency times of different doses during 90 min of the experiment showed similar pattern and temporal variation, however,the interaction of doseswas significant for the doses of 1 to 200 mg/Kg. **p < 0.01 compared to control B) %MPE_60_ of 100 mg/Kg of pregabalinthat achieved maximum level of antinociception. %MPE_75_ of 10, 100, 200 mg/Kg was increased compared to the control.*p < 0.05, **p < 0.01 and ***p < 0.005 compared to the control. C) AUC of 100 mg/Kg of pregabalin was increased compared to the control. *p < 0.05 and **p < 0.01 compared to the control. Data were expressed as mean ± SEM of 8 mice

The time course of antinociceptive effect of pregabalin at doses of 1, 10, 100, 200 and 400 mg/Kg increased immediately after the injection, but the highest latency times were achieved in the 60^th^ and 75^th^ min. The dose of 100 mg/Kg of pregabalin was the most effective. The temporal variations of latency time and pattern among different doses were not different (F_5,245_=0.81, p=0.5), but the interaction between dose and time was observed (F_25,245_=2.26, p=0.001). The latency times of control group significantly differed compared to doses of 10 and 100 mg/Kg (p < 0.01). No difference was observed between the controls compared to 400 mg/Kg (Figure 3A). 

Similar to Tramadol, maximal possible effect (%MPE) was achieved at the 60^th^ and 75^th^ minutes for all doses of pregabalin. At doses of 10, 100 and 200 mg/Kg the %MPE_75_ increased significantly compared to the control group (p < 0.05, p < 0.01 and p < 0.05). However, the maximum effect was seen at dose of 100 mg/Kg after 60 minutes (%MPE_60_ = 35.23±3.7%) that showed significant increase (p < 0.005) compared to %MPE_60_ of the control group (7.1+2.5%). %MPE_60_ of 100 mg/Kg of pregabalin was apparently more than that of 50 mg/Kg of tramadol (%MPE_60_ = 30.31+4.1%) (Figure 3B). The AUC of doses of 10, 100 and 200 mg/Kg of pregabalin also increased significantly compared to that of control group (10 and 200 mg/Kg; p<0.05 and 100 mg/Kg; p < 0.001) (Figure 3C). 

Dose-response relationship was observed neither in terms of %MPE nor in AUC. Based on curve estimation model with dependent variable %MPE, none of the functions provided r^2^> 0.7. Thus thedose of 10 mg/Kg of pregabalin (%MPE_75_=20.3± 5.2% and %MPE_60_=18.4± 4.4%), as the lowest effective dose which was carefully chosen for the study of combinations groups (Figure 3C).

*Antinociceptive effect of combination groups*

**Figure 4:**
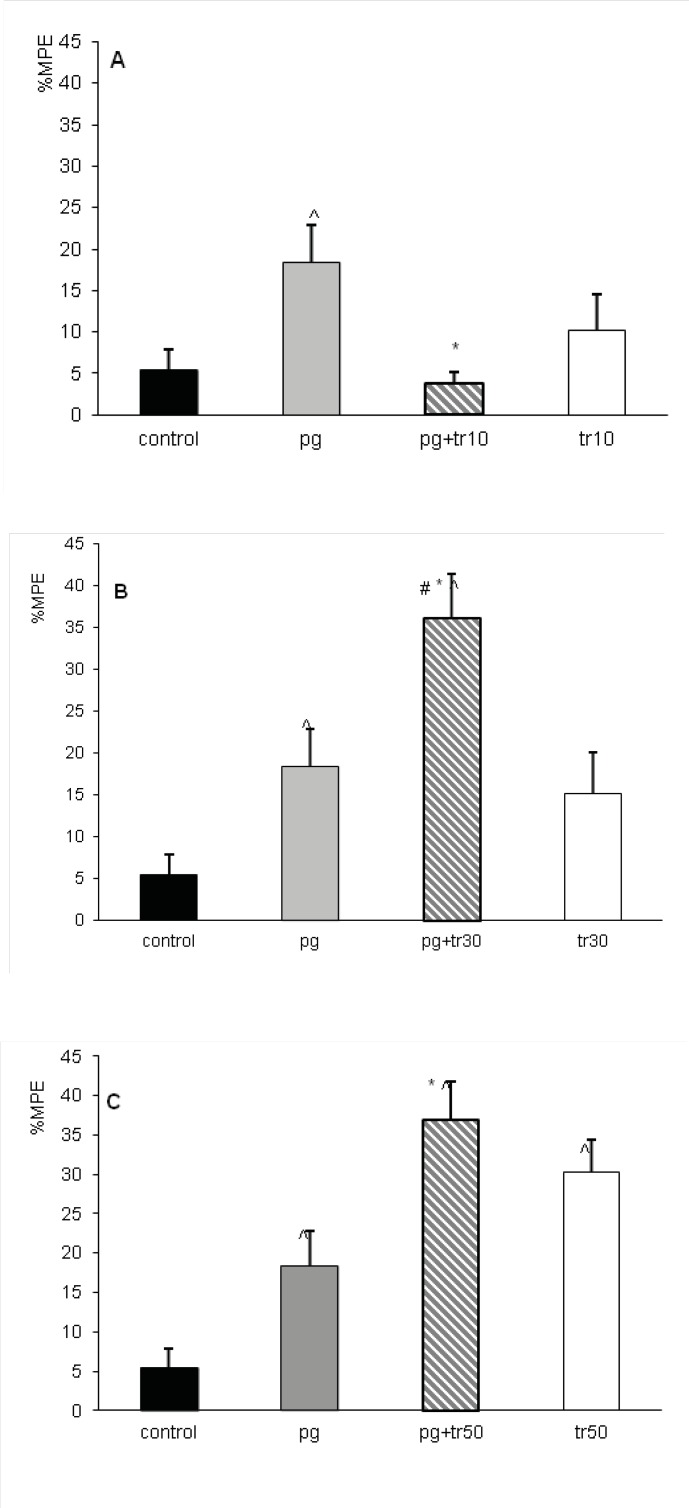
The antinociception of combination groups of pregabalin and tramadol in terms of maximum possible effect (%MPE_60_). Mice received intraperitoneally, normal saline (control n=12), the dose of 10 mg/Kg of pregabalin (pg) or tramadol at doses of 10 (tr10), 30 (tr30) and 50 mg/Kg (tr50) in combination groups of (pg+tr10), (pg+tr30) and (pg+tr50). A) %MPE_60_ of combination group (pg+tr10) decreased compared to pregabalin alone. B) %MPE_60_ of combination group (pg+tr30) increased compared to pregabalin, tramadol and control. C) %MPE_60_ of combination group (pg+tr50) increased compared to pregabalin and control. ^p < 0.05 compared to the control, *p < 0.05 compared to pregabalin, #p < 0.05 compared to tramadol. Data were expressed as mean ± SEM of 8 mice

Antinociceptive effect of the co-administration of tramadol at doses of 10, 30 and 50 mg/Kg with pregabalin at the dose of 10 mg/Kgwas assessed in combination groups; pg+tr10, pg+tr30 and pg+tr50. In combination groups, the maximum effect was achieved approximately after 60 minutes (%MPE_60_), so the comparisons were made measuring %MPE_60. _When tramadol and pregabalin were co-administrated at the dose of 10 mg/Kg, %MPE_60_ decreased significantly (p < 0.05) compared to the administration of pregabalin alone (Figure 4A). While co-administration of sub-analgesic dose of 30 mg/Kg of tramadol with 10 mg/Kg of pregabalin increased significantly the %MPE_60_ of combination group of (pg+tr30) compared to either tramadol or pregabalin (p<0.05) (Figure 4B). The %MPE_60_ of this group (36± 4.8%) also significantly increased compared to that produced by 40 mg/Kg of tramadol (%MPE_60_=17.2± 3.7% asshownin Figure 1B)(p<0.05) and even no significant difference was observed with that of 50 mg/Kg of tramadol (%MPE_60_=30.31± 4.1%). 

Increasing the dose of tramadol to 50 mg/Kg in combination group of (pg+tr50) did not increase its %MPE_60_ compared to tramadol; however, it continued to be significantly (p < 0.05) more than %MPE_60_ of pregabalin and the control groups (Figure 4C).

## Discussion

The results of this study showed the antinociceptive effect of pregabalin in tail flick test for the first time. The latency time increased from post injection time and the peak effect was observed at the 60^th^ and 75^th^ minutes. Maximum effect observed by 100 mg/Kg of pregabalin in terms of %MPE_60_, was comparable to%MPE_60 _of 50 and 80 mg/Kg of tramadol. The antinociception in this acute model of pain was not dose-dependent, although initially it increased as the dose was increased;however, subsequently, the antinociceptive effect reduced to the controlled levels. The decreased time response curve in tail flick test or the reduction of antinociceptive effect of pregabalin seen at 400 mg/kg could be due to its toxicity effect seen also in human clinical setting (3, 6, 10). Effectively, the highest dose used in previoussimilar studies of pain assessment was 100 or at maximum 200 mg/kg in hot plate test (7, 21-22, 24-27). Analgesic effect of pregabalin above these doses probably presents the same anomalies like decreased antinociceptionwhich we have also reportedin our study, although the nature of reflexive response mechanism of tail-flick may also be responsible. The explanation of this bimodal activity will be possible only after furtherstudies on pharmacokinetics and site of action of pregabalin.

It can be expected that the antinociceptive effect of 100 and 200 mg/Kgdoses of pregabalin are likely to decrease locomotor activity of mice. As well, it has been reported that pregabalin, at the doses over 100 mg/Kg (SC) or 200 mg/Kg (p.o.), significantly decreases the time spent on rota-rod in other rodents such as rats (21, 27). However, this wasnot of interest inour study and we considered that the reduction of locomotor activity did not affect antinociceptive assessment in this model of acute pain. Since tail flick response is not largely influenced by light anesthesia and also the dose in which the locomotion should be drastically reduced (400 mg/Kg), none of the antinociception indexes increased. 

The commencement of antinociception of pregabalin in this study occurred almost one hour after the injection (IP). The time to reach maximum antinociceptive effect is very different in other experimental models of pain. In inflammatory pain stimulated by formalin and in allodynia induced by trinitrobenzene sulfonic acid in rats, the peak effect of pregabalin happened within thirty minutes (21, 24). While, in colorectal distention model of visceral pain and in allodynia induced by the chronic constriction injury in mice, the peak result was achieved in one hour (25,28). Interestingly, antihyperalgesic activity of pregabalin induced by carrageenan in rats took two hours to happen (7) and the effect of oral doses lasted for four hours (27). Finally, in hot-plate model of acute pain which is the nearest model to tail flick test, maximum peak effect was achieved atthe 60^th^ min which is comparable to the results of this study (22). All these inconsistencies among peak effects obviously depended on the assumed method, species, route of administration and procedure rather than curve of plasma concentration for pregabalin which holds maximum concentration level at either 0.5 or 2 h in rats (27). 

Other researchers implementing different models of experimental pain, recommended similar dose range of pregabalin. Pregabalin reduced nociceptive scores in the second phase of inflammatory pain and like our study, this effect occurred atdoses of 10, 30 to 100 mg/Kg (7, 21, 25). The effective dose range used in our study is in complete agreement with previous studies that have investigated the inhibitory effect of pregabalin on colorectal distention response and also on declining colonic nociceptive threshold of visceral pain in sensitized rat (24, 27-28).

The general beliefpointed out for the effectiveness of gabapentinoids on pathological pain associated with central sensitizationsuch as neuropathic, postoperative or inflammatory pain while having no antinociceptive effect in acute physiological pain (3, 25, 29-30). Nonetheless, gabapentin, the prototype of gabapentinoids, showed antinociceptionin acute pain model of tail flick test and in acetic acid induced visceral pain in intact nervous system (16, 31). It was clearly shown in this study that the antinociception produced by 100 mg/Kg of pregabalin (%MPE_60_=35%) is almost similar to that of 50 mg/Kg of tramadol (%MPE_60_= 30%). Thus, our result and that of the recent study on hot plate (16, 22, 31) set forth that pregabalin is likely to have antinociceptive effect on transient model of pain in non-sensitized nervous system, even though dose-dependency of this effect has not been observed on a regular basis.

Unlike pregabalin, tramadol produced an evident dose-dependent effect in tail flick test at dose ranges of 10 to 80 mg/Kg as shown in time response curve, %MPE_75,60_ and AUC. The induced dose-dependent antiociceptive effect of tramadol was discussed in previous studies using different models of acute pain such as hot-plate, visceral pain induced by acetic acid and the inflammatory pain induced by formalin (11, 18-19).The results of our study particularly confirm to the results of another study, in which tramadol increased latency time in tail flick in a dose-dependent manner and also the calculated ED_50_which was 59.2 mg/Kg (32). In accordance, based on previous studies, tramadol produces dose-dependent antinociception at doses of 5 to 40 mg/Kg in rats (33) and similarly the sub-analgesic dose of 10 mg/Kg has no effect on pain threshold (34). 

The antinociceptive effect of combination treatment of pregabalin with tramadol varied using different doses of tramadol. The %MPE_60_ of combinational group of pg+tr10 decreased when non-analgesic dose of tramadol was added to the sub-analgesic dose of pregabalin;however, in combination group of pg+tr30, the interaction seemed to be supra-additive and in case of combination group ofpg+ tr50, it was nearly additive (Figure 4) .

No simple explanation exists for the interaction between pregabalin and tramadol or for the variation of interaction especially when different mechanisms account for each drug. The proportion of combined drugs might change the interaction from antagonism to synergism especially when a drug has multimodal mechanism. Indeed, in the combination of tramadol and dexketoprofen, only with potency ratio of 1:1 the synergisms was verified and with any other proportion for the combination this result was not evident (19).

The increased antinociceptive effect occurred in the combination groups of (pg+tr30) and (pg+tr50) can be explained by the complementary action of pregabalin and tramodol. It has been well established that the main mechanism of anticonvulsant and analgesic action of pregabalin is through binding to alpha-2-delta subunits of voltage dependent Ca^+2^ channels decreasing the release of norepinephrine, serotonin and dopamine and particularly glutamate (10,25-26,29). Since the antagonists of NMDA glutamate receptor enhances mu opioid antinociception (35), one can deduce that pregabalin by decreasing glutamate release (29) can increase the thermal antinociception of tramadol. There are other neuromodulators involved in pain transmission that are influenced by tramadol and pregabalin. Indeed, pregabalinreducessubstance P after inflammation in spinal cord slices (30), and reducesthe formalin- induced release of glutamate in spinal cord of dorsal horn (26). Whereas, tramadol decreasesthe effect of glutamate and substance P by inhibiting both ionotropic and metabotropic glutaminergic receptors (36). This additive effect (Figure 4B, C) was similarly reported in tail flick test in mice when gabapentin (prototype) at dose of 15 mg/Kg(%MPE =10% which is equal to %MPE produced by 10 mg/Kg of pregablin) enhanced the antinociception of tramadol (32).The synergistic interaction between tramadol and gabapentin has been proved previously using isobolographic study in inflammatory pain model in rats (11). 

However the analgesic effect of these two drugs in combination therapy depends also on the level of the effect produced by each drug. Indeed the interaction of pregabalin with tramadol changed to antagonism when non analgesic dose of tramadol (10 mg/ Kg) that produced 15% of effect was co-administrated in (prg+tr10) group (Figure 4A). Same results were observed in the combination of metamizol and tramadol. At low level of antinociception (below 25%) produced by each drug, the interaction was antagonistic while at 50% effect or higher all combinations resulted supra-additive (37). Another hypothesis in this regard might be the concurrent effect of tramadol and pregabalin on decreasing substance P and glutamate effect on pain transmission mentioned above (26, 30), occurringin a dose-dependent manner (36). Since this positive effect of tramadol happens above 50 mg/Kg, probably the dose of 10 mg/Kg of tramadol used in this study was insufficient for theinhibition of glutaminergic receptors. Therefore, non analgesic dose of tramadol was not able to increase the antinociceptive effect in prg+tr10 group. Other pathways and receptors might explain this non-enhanced antinociceptive effect in prg+tr10 group. For example; alpha (2) antagonists increased the antinociception of tramadol in tail flick test of mice while the antinociception of pregabalin was decreased by same agents removing the inhibitory influence of locus coeruleus after nerve injury (38-39). 

In conclusion, our results demonstrated that pregabalin is likely to have comparable antinociceptive effect to tramadol even though no dose dependent relationship was observed similar totramadol. Withregard to interaction between tramadol and pregabalinand contrary to general beliefthat lower dose of each drug produces lesser side effect and is preferable in combination therapy,the addition of analgesic doses of tramadol increases the antinociceptive effect while non analgesic doses reduced the antinociceptive effect in combined groups. Thusin these multimodal analgesics the effect of combination changes with ratio and level of the effect produced, the fact that is relevant when attempting to use their combination in pain treatment. Thus, more caution should be paid in use of tramadol and pregabalin at very low doses in clinical practice. Isobolographic study will add valuable information in interpreting the statement of this combination, and also more investigations are recommended to establish the modality and correct proportion of co-administration for clinical applications. 
